# Localizable entanglement as a necessary resource of controlled quantum teleportation

**DOI:** 10.1038/s41598-018-33185-5

**Published:** 2018-10-12

**Authors:** Artur Barasiński, Ievgen I. Arkhipov, Jiří Svozilík

**Affiliations:** 1 RCPTM, Joint Laboratory of Optics of Palacký University and Institute of Physics of CAS, Faculty of Science, Palacký University, 17. Listopadu 12, 771 46 Olomouc, Czech Republic; 2 Institute of Physics, University of Zielona Góra, Z. Szafrana 4a, 65-516 Zielona Góra, Poland; 3 Quantum Optics Laboratory, Universidad de los Andes, A.A. 4976 Bogotá, D.C. Colombia; 4 Centro de Física Aplicada y Tecnología Avanzada, Universidad Nacional Autónoma de México, Boulevard Juriquilla 3001, 76230 Juriquilla, Querétaro Mexico

## Abstract

We analyze the controlled teleportation protocol through three-qubit mixed states. In particular, we investigate the relation between the faithfulness of the controlled teleportation scheme and entanglement. While our knowledge concerning controlled teleportation and entanglement in pure states is well established, for mixed states it is considerably much harder task and very little has been done in this field. Here, we present counterintuitive results that provide a new light on controlled teleportation protocol. It is shown that even mixed biseparable states are useful for this protocol along with genuine entangled three-qubit states.

## Introduction

Quantum entanglement is one of the striking features of quantum systems that makes them different from their classical counterparts^1,2^. Entanglement plays a central role for numerous quantum information protocols that are used in one-way quantum computation^3^, quantum communication^4^ and quantum cryptography^5^, to name a few. The more a quantum state is entangled, the better it will perform in the information processing and communication tasks, compared to any unentangled state^6,7^. However, the question if entanglement is a necessary resource for quantum protocols remains still open.

One of the most important applications of entanglement is a process of quantum teleportation^8^. In a standard protocol of a single qubit state teleportation only two parties are involved^8^, namely, the sender and receiver who share a maximally entangled two-qubit Bell state (maximally entangled channel) in advance. In the first step, the sender performs a two-qubit measurement in the Bell-state basis on one qubit from the teleportation channel and the additional qubit which state wishes to be teleported. Based on the measurement outcome, the receiver uses appropriate unitary operations on the remaining qubit from the Bell pair to perfectly reconstruct the state to be teleported. In the ideal quantum teleportation procedure, the state can be recovered with fidelity *F* = 1, while the faithfulness of the teleportation attainable by a purely classical channel cannot exceed $F=\frac{2}{3}$^9,10^. In general, the lower and upper bound of teleportation fidelity for a single copy of a two-qubit (mixed) channel with a given amount of entanglement writes as^11^

1 $${\rm{\max }}\{\frac{3+{\mathscr{C}}}{6},\frac{1+2{\mathscr{C}}}{3}\}\le F\le \frac{2+{\mathscr{C}}}{3},$$

where ${\mathscr{C}}$ stands for the bipartite concurrence^12^ and the upper bound is reached by pure two-qubit states. These relations also provide a clear manifestation of quantum entanglement as a necessary resource to execute the standard teleportation protocol within the quantum limit faithfulness. Naturally, the inverse relation is not true, since not every mixed entangled state can reach quantum fidelity^13^.

Over time, quantum teleportation has been studied and developed in numbers of ways such as multipartite systems^14,15^. In particular, a tripartite variant of quantum teleportation called controlled quantum teleportation (CQT) has been proposed by Karlsson and Bourennane^16^. For this scenario, the success or failure of teleportation process is determined by a controller, i.e. an unknown state of a single qubit can be teleported from sender to receiver with fidelity ${F}_{CQT}\ge \frac{2}{3}$ only with the permission of the controller. Without controller’s participation the teleportation fidelity (henceforth referred to as the non-conditioned fidelity *F*_*NC*_) is no better than the fidelity of a classical channel, ${F}_{NC}\le \frac{2}{3}$^17,18^. Here, the question whether entanglement is a necessary resource for CQT is much more sophisticated. From one hand, it is because the tripartite scenario offers a richer variety of different types of entanglement distinguished with respect to stochastic local operations and classical communication (SLOCC). Specifically, a pure three-qubit state can either be completely separable, biseparable or tripartite entangled^19^. Furthermore, there are two locally inequivalent classes of tripartite entanglement, namely the GHZ-class and the W-class. On the other hand, the analysis of the relation between entanglement and CQT protocol should be restricted to such classes of states which provide the controller’s authority.

In a pure-state regime a direct relation between entanglement and the maximal fidelity of tripartite CQT is given by^20^

2 $${F}_{CQT}(\psi )=\frac{2+\sqrt{\tau (\psi )+{{\mathscr{C}}}_{S,R}^{2}(\psi )}}{3},$$

where |*ψ*〉 is an arbitrary three-qubit pure state, *τ*(*ψ*) denotes the three-tangle^21^ (a tripartite entanglement monotony) and ${{\mathscr{C}}}_{S,R}(\psi )={\mathscr{C}}({\rho }_{S,R})$ is the bipartite concurrence for *ρ*_*S*,*R*_ = Tr_*C*_|*ψ*〉 〈*ψ*|. The subscripts *C*, *R* and *S* represent the qubits of controller, receiver and sender, respectively. Based on the above-mentioned concept of CQT, the teleportation fidelity *F*_*CQT*_ in Eq. (2) is meaningful if and only if the non-conditioned fidelity ${F}_{NC}({\rho }_{S,R})\le \frac{2}{3}$ what implies immediately that none of pure separable and pure biseparable states are useful for CQT. The CQT protocol has been successfully investigated via several classes of partially tripartite entangled pure states^16–18^, in particular the generalized GHZ states. Therefore, the tripartite entanglement can be considered as a necessary resource of CQT^15,18,22,23^.

Nevertheless, in any realistic implementation of the protocol, various kinds of noise are inherently present and such conditions result in the reduction of entanglement of the quantum channels. Although a considerable research has been devoted to analyze tripartite teleportation through noisy channels^24–27^, much less attention has been paid to discuss the relation between noisy CQT protocol and entanglement (especially the entanglement classification). Therefore, there are several important questions that emerge: What kind of entanglement, classified with respect to SLOCC, is truly needed for CQT? What kind of mixed three-qubit states is useful for CQT? Is every mixed state that can be expressed as a mixture of product or biseparable states unsuitable for CQT?

We recall that the entanglement classification of pure states can be extended to mixed states by considering the classes of all pure states in the convex decomposition of state under consideration^28^. From the operational point of view the mixed state entanglement classes can be distinguished by various entanglement measures: A state is GHZ-type entangled iff the three-tangle^21^ does not vanish, *τ* > 0. A state is W-type entangled iff the genuine multipartite concurrence ${{\mathscr{C}}}_{GME}$^29,30^ does not vanish, but the three-tangle does, *τ* = 0 and ${{\mathscr{C}}}_{GME} > 0$. For biseparability, an appropriate measure is the convex roof of the square root of the global entanglement^31^. For unentangled states all entanglement measures vanish.

Motivated by all these remarks, here we investigate the performance of CQT through mixed-state channels and discuss its relation with various kind of entanglement. In particular, we calculate CQT for two representative tripartite mixed states, namely the GHZ-symmetric and *X*–matrix states. We show that (in contrast to pure-state channels) tripartite entanglement is not a necessary resource of CQT for mixed states and classical correlation are sufficient to ensure the ability to control the teleportation protocol. We find the upper and lower bounds of *F*_*CQT*_ which are satisfied by any state useful for CQT, for a general n-qubit state. The boundaries presented in this paper are directly analogous to the two-qubit fidelity-entanglement relationship given by Eq. (1). We also discuss further interesting properties of *F*_*CQT*_ based on the convex decomposition of the tripartite mixed state.

## Controlled Teleportation Protocol

We first review the protocol of CQT which is a variation of the splitting and reconstruction of quantum information over the GHZ state proposed by Hillery *et al*.^32^ and present its extension to n-qubit state.

For this purpose, let *ρ* be a tripartite state (channel) with distinct parties *C*, *R*, *S* = {1, 2, 3}, respectively. Then, the teleportation scheme over such three-qubit state can be described as follows: (i) The controller makes a one-qubit orthogonal measurement on the subsystem *C* with an outcome *t*; (ii) The sender prepares an arbitrary one-qubit state, and then makes a two-qubit orthogonal measurement on the one qubit and the subsystem *S*; (iii) The receiver applies on the subsystem *R* proper unitary operations related to the 3-bit classical information of the two above measurement results. For such scenario the total fidelity *F*_*CQT*_ of tripartite state *ρ* can be written as an average value over the teleportation fidelity through the reduced state ${\rho }_{SR}^{t}$ what can be expressed in a general form^20^

3 $${F}_{CQT}(\rho )=\mathop{{\rm{\max }}}\limits_{{U}_{C}}\,[\sum _{t=0}^{1}\,\langle t|{U}_{C}{\rho }_{C}{U}_{C}^{\dagger }|t\rangle F({\rho }_{SR}^{t})]$$

where

4 $${\rho }_{SR}^{t}=\frac{{{\rm{Tr}}}_{C}[{U}_{C}^{\dagger }|t\rangle \langle t|{U}_{C}\otimes {11}_{4}\rho {U}_{C}^{\dagger }|t\rangle \langle t|{U}_{C}\otimes {11}_{4}]}{\langle t|{U}_{C}{\rho }_{C}{U}_{C}^{\dagger }|t\rangle }$$

is the resulting state of the joined subsystem *SR* after the local measurement on the subsystem *C* with the measurement outcome *t* and $\langle t|{U}_{C}{\rho }_{C}{U}_{C}^{\dagger }|t\rangle$ denotes the probability of receiving the outcome *t* within one-qubit measurement. Here, *U*_*C*_ is a 2 × 2 unitary matrix, ${11}_{4}$ stands for an 4 × 4 identity matrix and *ρ*_*C*_ = Tr_*SR*_(*ρ*) is a reduce one-qubit state. Finally, the quantity $F({\rho }_{SR}^{t})$ corresponds to the faithfulness of the two-qubit teleportation through the resulting state ${\rho }_{SR}^{t}$. It is known that for a standard teleportation protocol the maximal achievable fidelity is given by $F(\rho )=\frac{2f(\rho )+1}{3}$, where *f*(*ρ*) = max_*e*_ 〈*e*|*ρ*|*e*〉 is the fully entangled fraction^33,34^. Using this expression, one can express both fidelities in a compact form as

5 $${F}_{NC}(\rho )=\frac{2{f}_{NC}(\rho )+1}{3},\,{\rm{where}}\,{f}_{NC}(\rho )=f({{\rm{Tr}}}_{C}\rho ),$$

6 $${F}_{CQT}(\rho )=\tfrac{2{f}_{CQT}(\rho )+1}{3},\,{\rm{where}}\,{f}_{CQT}(\rho )=\mathop{{\rm{\max }}}\limits_{{U}_{C}}\,[\sum _{t=0}^{1}\,\langle t|{U}_{C}{\rho }_{C}{U}_{C}^{\dagger }|t\rangle f({\rho }_{SR}^{t})].$$

It is worth mentioning that for an arbitrary pure state, *ρ* = |*ψ*〉 〈*ψ*|, Eq. (6) can be further simplify and written in the form given by Eq. (2) ^20^.

Finally, we note that the CQT protocol can also be extended to n-qubit case. Then, the conditional fidelity in Eq. (3) takes almost identical form of ${F}_{CQT}(\rho )={{\rm{\max }}}_{{U}_{C}}\,[{\sum }_{t=0}^{k}\,\langle t|{U}_{C}{\rho }_{C}{U}_{C}^{\dagger }|t\rangle F({\rho }_{SR}^{t})]$, where *k* = 2^*n*−2^ − 1 and *U*_*C*_ is a 2^*n*−2^ × 2^*n*−2^ unitary matrix. The shape of matrix *U*_*C*_ depends on the strategy chosen by the controller. In particular, when the controller’s strategy is based on *n* − 2 local measurement the unitary matrix ${U}_{C}={U}_{C}^{{j}_{1}}\otimes {U}_{C}^{{j}_{2}}\otimes \cdots \otimes {U}_{C}^{{j}_{n-2}}$, where ${U}_{C}^{j}$ corresponds to 2 × 2 unitary matrix and indices $\{{j}_{1},\ldots ,{j}_{n-2}\}\subset \{1,\ldots ,n\}$. In the same way one should interpret the Eq. (4).

## Results

### Controlled teleportation via GHZ-symmetric states

**Figure 1:**
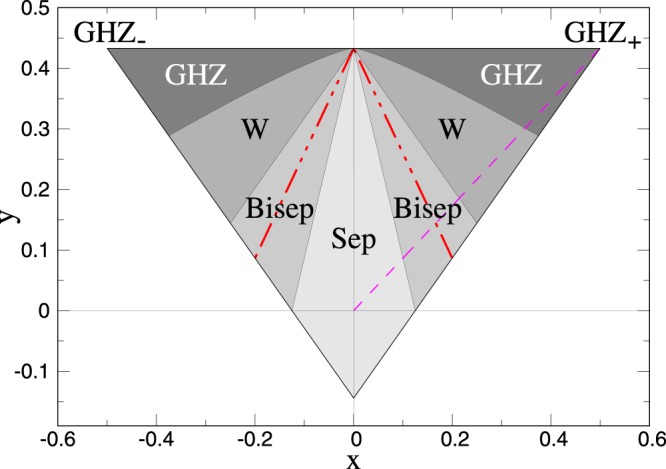
The diagram of the SLOCC entanglement classes of the GHZ-symmetric states^36^: separable class (Sep), biseparable class (Bisep), W class and GHZ class. The dashed-dotted line represents Eq. (11), i.e. the lower border of *ρ*^*GS*^ states useful for CQT. The upper corners depict the pure states *GHZ*^+^ and *GHZ*^−^ and the dashed line corresponds to the Werner states (*ρ*^*WS*^).

Let us first consider a particular family of mixed states which have recently received a lot of attention, namely GHZ-symmetric states^35,36^. This family contains all tripartite mixed states, invariant under the following symmetries ${\mathscr{U}}$: qubit permutation, application of ${\sigma }_{x}\otimes {\sigma }_{x}\otimes {\sigma }_{x}$ (i.e. simultaneous three-qubit flips) and simultaneous (local) phase rotations of the form

7 $${U}^{GS}({\phi }_{1},{\phi }_{2})={e}^{i{\phi }_{1}{\sigma }_{z}}\otimes {e}^{i{\phi }_{2}{\sigma }_{z}}\otimes {e}^{-i({\phi }_{1}+{\phi }_{2}){\sigma }_{z}},$$

where *σ*_*x*_ and *σ*_*z*_ are the Pauli operators. The general form of three qubits GHZ-symmetric states can be written as

8 $${\rho }^{GS}(x,y)=(\tfrac{2y}{\sqrt{3}}+x)|GH{Z}^{+}\rangle \langle GH{Z}^{+}|+(\tfrac{2y}{\sqrt{3}}-x)|GH{Z}^{-}\rangle \langle GH{Z}^{-}|+\tfrac{\sqrt{3}-4y}{8\sqrt{3}}{11}_{8},$$

where $|GH{Z}^{\pm }\rangle \equiv \frac{|000\rangle \pm |111\rangle }{\sqrt{2}}$ and ${11}_{8}$ stands for an 8 × 8 identity matrix. In order to satisfy the positive semidefinite requirement, *ρ*^*GS*^(*x*, *y*) ≥ 0, the coordinates *x* and *y* are limited by −$\frac{1}{4\sqrt{3}}\le y\le \frac{\sqrt{3}}{4}$ and $|x|\le \frac{1}{8}+\frac{\sqrt{3}}{2}y$. Any point inside this triangle represents a GHZ-symmetric state. For this family several entanglement classes with respect to SLOCC can be distinguished^35,36^. Specifically, the GHZ-class which is limited from the bottom by the parametrized curve $\{{x}^{W},{y}^{W}\}=\{\frac{{v}^{5}+8{v}^{3}}{8(4-{v}^{2})},\frac{\sqrt{3}}{4}\frac{4-{v}^{2}-{v}^{4}}{4-{v}^{2}}\}$, where −1 ≤ *v* ≤ 1 and turns into the W-class (cf. Fig. 1). Such parametrized curve is often referred to as the GHZ-W line. The lower bound of the W-class is certified by vanishing of ${{\mathscr{C}}}_{GME}({\rho }^{GS}(x,y))=\,{\rm{\max }}\,\{0,-\,\frac{3}{4}+2|x|+\sqrt{3}y\}$. This line separates W-class and the biseparable states (hereafter the W-B line). Finally, the biseparable states are restricted by $y=\frac{\sqrt{3}}{4}-2\sqrt{3}|x|$.

It is well-known that in a pure-state regime the generalized GHZ state and a group of W-class states^19^ are useful for controlled teleportation^18^. In order to verify whether mixed states which belong to GHZ-class and W-class are also suitable for CQT both fidelities, given by Eqs (5) and (6), need to be determined. In order to do this, we use the Horodecki theorem^13^ and apply the general form of 2 × 2 unitary matrix *U*_*C*_ in Eq. (6). Then, after straightforward optimization we have found that

9 $${F}_{NC}({\rho }^{GS})=\frac{1}{18}[9+4\sqrt{3}y],$$

10 $${F}_{CQT}({\rho }^{GS})=\frac{1}{6}[3+4|x|+\frac{4\sqrt{3}}{3}y],$$

where our calculations are restricted to the three-qubit configuration *C* = 1, *S* = 2 and *R* = 3 as mentioned before. Note that such assumption can be done without loss of generality, since all GHZ-symmetric states and hence, Eqs (9) and (10) are invariant under qubit permutation.

Based on Eq. (9) and the limitation of coordinate *y*, it is clear that $\frac{5}{9}\le {F}_{NC}({\rho }^{GS})\le \frac{2}{3}$ in the entire range of {*x*, *y*} and hence the faithfulness of teleportation performed without controller permission cannot exceed the classical limit which fulfills the first criterion of CQT. This result can be easily explained since ${\rho }_{S,R}^{GS}={{\rm{Tr}}}_{C}{\rho }^{GS}$ is a diagonal matrix and hence, it can be decomposed into a convex combination of product states, i.e. ${{\mathscr{C}}}_{S,R}({\rho }_{S,R}^{GS})=0$. On the other hand, from Eq. (10) one can find that ${F}_{CQT}({\rho }^{GS}) > \frac{2}{3}$ if

11 $$y > {y}_{Q}(x)=\frac{\sqrt{3}}{4}-\sqrt{3}|x|.$$

In other words, any state *ρ*^*GS*^(*x*, *y*) with given *x* and *y* > *y*_*Q*_(*x*) is suitable for CQT within the quantum limit. By comparing *y*_*Q*_ with the borders of the SLOCC entanglement classes one can find that CQT can be perform not only through the mixed states that belong to the GHZ- and W-class but, surprisingly, also via the mixed biseparable states. As we see in Fig. 1, the *y*_*Q*_ curve is below the W-B line. This result is very intriguing because there is no pure biseparable state useful for CQT and therefore, this implies several consequences. First, since CQT can be performed without any type of tripartite entanglement, it cannot be considered as a necessary resource of this protocol and the controller’s capability is not ensured by the tripartite entanglement as it is commonly thought^22^. Furthermore, the usefulness of biseparable GHZ-symmetric states for CQT clearly shows that one cannot estimate *F*_*CQT*_ of mixed states based on the “predefined” entanglement properties such as *τ*(*ρ*) and ${{\mathscr{C}}}_{S,R}(\rho )$. In order to emphasize this fact, we note that outside the GHZ- and W-class both $\tau ({\rho }^{GS})={{\mathscr{C}}}_{GME}({\rho }^{GS})=0$ and the bipartite concurrence ${{\mathscr{C}}}_{S,R}={\mathscr{C}}({\rho }_{S,R}^{GS})=0$. As a result of Eq. (2) one should expect ${F}_{CQT}=\frac{2}{3}$ while the maximal fidelity for the biseparable GHZ-symmetric states is equal to $\frac{13}{18}$. Finally, it is known that entanglement, in particular tripartite entanglement, vanishes in the presence of noise. Therefore, the performance of CQT through biseparable states suggests that the controlled teleportation can be less fragile against noise than for the tripartite entanglement. In order to illustrate this, let us discuss a special case of GHZ-symmetric family, namely the three-qubit Werner states (WS) which can be considered as a global depolarizing noise that brings the GHZ state to the completely unpolarized state

12 $${\rho }^{WS}(p)={\rho }^{GS}(\tfrac{(1-p)}{2},\tfrac{\sqrt{3}(1-p)}{4})=(1-p)|GH{Z}^{+}\rangle \langle GH{Z}^{+}|+\tfrac{p}{8}{11}_{8},$$

where 0 ≤ *p* ≤ 1 is a probability of replacing the GHZ state with a completely mixed state ${11}_{8}/8$. The three-qubit Werner states can be considered as an imperfect preparation of the quantum channel. In this case, the fidelity is given by ${F}_{CQT}({\rho }^{WS})=\frac{2-p}{2}$ while the genuine tripartite concurrence ${{\mathscr{C}}}_{GME}({\rho }^{WS})=\,{\rm{\max }}\,\{0,1-\frac{7}{4}p\}$. From these two expressions it is easy to find that the *F*_*CQT*_(*ρ*^*WS*^) falls into the classical teleportation limit when ${p}_{F}=\frac{2}{3}$ while the tripartite entanglement vanishes for ${p}_{T}=\frac{4}{7} > {p}_{F}$. Interestingly, when the regime of biseparable states is entered then the corresponding fidelity *F*_*CQT*_(*ρ*^*WS*^(*p*_*T*_)) ≈ 0.71. This value is around 4% greater than the classical protocol limit. The last remark has a particular experimental meaning, where the measured results can be masked by the statistical fluctuations which are naturally present in any physical implementation of the protocol. Consequently, the difference between *F*_*CQT*_ and the classical protocol limit around 2–3% is required to be noticeable in the laboratory^37,38^.

As we see in Fig. 1, the line *y*_*Q*_ does not overlap with the lower bound of the biseparable states and hence not every biseparable mixed state can yield the quantum fidelity for CQT. In order to explain the nature of the “quantum” teleportation line in Eq. (11), let us (by analogy to Eq. (6)) determine the average concurrence between the sender and receiver after the optimal one-qubit orthogonal measurement on the subsystem *C*

13 $${{\mathscr{C}}}_{L}(\rho )=\mathop{{\rm{\max }}}\limits_{{U}_{C}}\,[\sum _{t=0}^{1}\,\langle t|{U}_{C}{\rho }_{C}{U}_{C}^{\dagger }|t\rangle {\mathscr{C}}({\rho }_{SR}^{t})].$$

where ${\rho }_{SR}^{t}$ has the same form as Eq. (4), ${\mathscr{C}}({\rho }_{SR}^{t})$ stands for bipartite concurrence of ${\rho }_{SR}^{t}$ and the optimization is performed over all 2 × 2 unitary matrices *U*_*C*_. Such quantity is known as a localizable concurrence^39,40^ (restricted to the projective von Neuman measurements) and in general the optimal matrix *U*_*C*_ in Eqs (6) and (13) is not the same one. We note that ${{\mathscr{C}}}_{L}(\rho )$ in Eq. (13) can be easily extanded to n-qubit system in the same way as we discussed for *F*_*CQT*_^39,40^.

Applying the localizable concurrence to the GHZ-symmetric states one can find that the optimal one-qubit orthogonal measurement on the subsystem *C* (i.e for *U*_*C*_ that maximize Eq. (13)) implies the reduction of the state *ρ*^*GS*^, with the probability $\langle t|{U}_{C}{\rho }_{C}{U}_{C}^{\dagger }|t\rangle =\frac{1}{2}$, to one of two channels,

$${\rho }_{SR}^{t}=(\begin{array}{cccc}\tfrac{1}{4}(1+\tfrac{4\sqrt{3}y}{3}) & 0 & 0 & {(-1)}^{t}x\\ 0 & \tfrac{1}{4}(1-\tfrac{4\sqrt{3}y}{3}) & 0 & 0\\ 0 & 0 & \tfrac{1}{4}(1-\tfrac{4\sqrt{3}y}{3}) & 0\\ {(-1)}^{t}x & 0 & 0 & \tfrac{1}{4}(1+\tfrac{4\sqrt{3}y}{3})\end{array}),$$

where *t* = 0, 1. Then, the average bipartite concurrence of *ρ*^*GS*^ is given by ${{\mathscr{C}}}_{L}({\rho }^{GS})=2\,{\rm{\max }}\,\{0,-\,\frac{1}{4}+|x|+\frac{y}{\sqrt{3}}\}$. As we see, ${{\mathscr{C}}}_{L}({\rho }^{GS})\ge 0$ if and only if *y* > *y*_*Q*_ for a given *x* what is consistent with Eq. (11). This means that *ρ*^*GS*^ is useful for CQT in the quantum limit if there exists the non-zero localizable entanglement between sender and receiver. Based on this observation, the fidelity *F*_*CQT*_(*ρ*^*GS*^) can be written as

14 $${F}_{CQT}({\rho }^{GS})\le \frac{2+{{\mathscr{C}}}_{L}({\rho }^{GS})}{3},$$

with the equality for *y* > *y*_*Q*_ (equivalently, for ${{\mathscr{C}}}_{L}({\rho }^{GS}) > 0$). This result can be further generalize as follows.

#### **Proposition 1**.

Given a mixed state of *n* qubits *ρ* with localizable concurrence ${{\mathscr{C}}}_{L}(\rho )$, then its fidelity of controlled teleportation *F*_*CQT*_ is bounded by

15 $${\rm{\max }}\{\frac{3+{{\mathscr{C}}}_{L}(\rho )}{6},\frac{1+2{{\mathscr{C}}}_{L}(\rho )}{3}\}\le {F}_{CQT}(\rho )\le \frac{2+{{\mathscr{C}}}_{L}(\rho )}{3}.$$

and the localizable concurrence is a necessary resource for controlled teleportation.

#### *Proof*.

Suppose that there is an unitary matrix ${\tilde{U}}_{C}$ which maximizes Eq. (3) (*k* = 1) or its n-partite extension (*k* = 2^*n*−2^ − 1) and ${\tilde{\rho }}_{SR}^{t}$ is an optimal two-qubit state given by Eq. (4) when ${U}_{C}={\tilde{U}}_{C}$. From Eq. (1) it is apparent that one can always expect $F({\tilde{\rho }}_{SR}^{t})\le \frac{2+{\mathscr{C}}({\tilde{\rho }}_{SR}^{t})}{3}$, where ${\mathscr{C}}({\tilde{\rho }}_{SR}^{t})$ is the bipartite concurrence of ${\tilde{\rho }}_{SR}^{t}$. Then, Eq. (3) yields

16 $$\begin{array}{rcl}{F}_{CQT}(\rho ) & = & \sum _{t=0}^{k}\,\langle t|{\tilde{U}}_{C}{\rho }_{C}{\tilde{U}}_{C}^{\dagger }|t\rangle F({\rho }_{SR}^{t})\\  & \le  & \sum _{t=0}^{k}\,\langle t|{\tilde{U}}_{C}{\rho }_{C}{\tilde{U}}_{C}^{\dagger }|t\rangle \tfrac{2+{\mathscr{C}}({\tilde{\rho }}_{SR}^{t})}{3}\\  & \le  & \mathop{{\rm{\max }}}\limits_{{U}_{C}}\,[\sum _{t=0}^{k}\,\langle t|{U}_{C}{\rho }_{C}{U}_{C}^{\dagger }|t\rangle \tfrac{2+{\mathscr{C}}({\rho }_{SR}^{t})}{3}].\end{array}$$

Further simplification of the right-hand side of Eq. (16) provides ${F}_{CQT}(\rho )\le \frac{1}{3}(2+{{\rm{\max }}}_{{U}_{C}}\,[{\sum }_{t=0}^{k}\,\langle t|{U}_{C}{\rho }_{C}{U}_{C}^{\dagger }|t\rangle {\mathscr{C}}({\rho }_{SR}^{t})])$, where we have used ${{\rm{\max }}}_{{U}_{C}}\,[{\sum }_{t=0}^{k}\langle t|{U}_{C}{\rho }_{C}{U}_{C}^{\dagger }|t\rangle ]={{\rm{\max }}}_{{U}_{C}}\,[{\rm{Tr}}({U}_{C}{\rho }_{C}{U}_{C}^{\dagger })]=1$. Moreover, the second term of this expression corresponds to the localizable concurrence given by Eq. (13). Therefore, Eq. (16) can be expressed as ${F}_{CQT}(\rho )\le \frac{2+{{\mathscr{C}}}_{L}(\rho )}{3}$.

Similarly, let us assume that there is an unitary matrix ${\tilde{U}}_{C}$ which maximizes the localizable concurrence Eq. (13). Then, Eq. (1) implies that ${\rm{\max }}\,\{\frac{3+{\mathscr{C}}({\tilde{\rho }}_{SR}^{t})}{6},\frac{1+2{\mathscr{C}}({\tilde{\rho }}_{SR}^{t})}{3}\}\le F({\tilde{\rho }}_{SR}^{t})$ and thus

17 $$\begin{array}{rcl}\sum _{t=0}^{k}\,\langle t|{\tilde{U}}_{C}{\rho }_{C}{\tilde{U}}_{C}^{\dagger }|t\rangle \,{\rm{\max }}\{\tfrac{3+{\mathscr{C}}({\tilde{\rho }}_{SR}^{t})}{6},\tfrac{1+2{\mathscr{C}}({\tilde{\rho }}_{SR}^{t})}{3}\} & \le  & \sum _{t=0}^{k}\,\langle t|{\tilde{U}}_{C}{\rho }_{C}{\tilde{U}}_{C}^{\dagger }|t\rangle F({\tilde{\rho }}_{SR}^{t})\\  & \le  & \mathop{{\rm{\max }}}\limits_{{U}_{C}}\,[\sum _{t=0}^{k}\,\langle t|{U}_{C}{\rho }_{C}{U}_{C}^{\dagger }|t\rangle F({\rho }_{SR}^{t})]\\  & = & {F}_{CQT}(\rho ).\end{array}$$

Based on Eq. (13) and the fact that ${{\rm{\max }}}_{{U}_{C}}\,[X\,{\rm{\max }}\,\{Y,Z\}]=\,{\rm{\max }}\,\{{{\rm{\max }}}_{{U}_{C}}\,[XY],{{\rm{\max }}}_{{U}_{C}}\,[XZ]\}$ the most left-hand side of Eq. (17) can be written as ${\rm{\max }}\,\{\frac{3+{{\mathscr{C}}}_{L}(\rho )}{6},\frac{1+2{{\mathscr{C}}}_{L}(\rho )}{3}\}\le {F}_{CQT}(\rho )$.$\square$

It should be highlighted that Eq. (15) has a similar form as Eq. (1) what implies that the localizable concurrence plays the same role in CQT as bipartite concurrence in the standard teleportation protocol. In fact, if *ρ* is biseparable with respect to controller’s qubit (i.e. of the form ρ = ρ_SR_⊗ρ_C_) then ${{\mathscr{C}}}_{L}(\rho )={\mathscr{C}}({{\rm{Tr}}}_{C}\rho )$ and Eq. (15) becomes equivalent to Eq. (1).

### Controlled teleportation via triqubit X-matrices

In this section, we investigate the existence of other mixed biseparable states suitable for CQT. In particular, we verify what is the maximal attainable fidelity *F*_*CQT*_ for such states. For this purpose we analyze the X-matrices of Yu and Eberly^41^ represented by a density matrix of three qubits, written in an orthonormal product basis, whose nonzero elements are only diagonal or antidiagonal. The *X*–matrix can be written than as

$${\rho }_{X}=(\begin{array}{llllllllll}{a}_{1} &  &  &  &  &  &  &  &  & {z}_{1}\\  & \cdot  &  &  &  &  &  &  & \cdot  & \\  &  & \cdot  &  &  &  &  & \cdot  &  & \\  &  &  & \cdot  &  &  & \cdot  &  &  & \\  &  &  &  & {a}_{4} & {z}_{4} &  &  &  & \\  &  &  &  & {z}_{4}^{\ast } & {b}_{4} &  &  &  & \\  &  &  & \cdot  &  &  & \cdot  &  &  & \\  &  & \cdot  &  &  &  &  & \cdot  &  & \\  & \cdot  &  &  &  &  &  &  & \cdot  & \\ {z}_{1}^{\ast } &  &  &  &  &  &  &  &  & {b}_{1}\end{array})$$

where $|{z}_{j}|\le \sqrt{{a}_{j}{b}_{j}}$ and ${\sum }_{j}\,({a}_{j}+{b}_{j})=1$ to ensure the positivity and normalization of *ρ*_*X*_. For the triqubit X-matrices the border between tripartite entangled and biseparable classes is determined by disappearance of ${C}_{GME}({\rho }_{X})=2\,{\rm{\max }}\,\{0,|{z}_{j}|-{\omega }_{j}\}$, where ${\omega }_{j}={\sum }_{k\ne j}\,\sqrt{{a}_{k}{b}_{k}}$ and 1 ≤ *k* ≤ 4. We note that the GHZ-symmetric states discussed in the previous section are special examples of *ρ*_*X*_, i.e. ${a}_{1}={b}_{1}=\frac{1}{8}(1+4\sqrt{3}y)$, ${a}_{2}=\cdots ={b}_{4}=\frac{1}{24}(3+4\sqrt{3}y)$ and *z*_1_ = *x*, *z*_2_ = *z*_3_ = *z*_4_ = 0.

Performing appropriate optimizations, one can find that both fidelities of triqubit X-matrices are given by

18 $${F}_{NC}({\rho }_{X})=\frac{3+|{{\rm{\Delta }}}_{1}|}{6},$$

19 $${F}_{CQT}({\rho }_{X})=\,{\rm{\max }}\,\{{F}_{CQT}^{(1)},{F}_{CQT}^{(2)},{F}_{CQT}^{(3)},{F}_{CQT}^{(4)}\},$$

where Δ_1_ = *a*_1_ − *a*_2_ − *a*_3_ + *a*_4_ + *b*_1_ − *b*_2_ − *b*_3_ + *b*_4_ and

20 $$\begin{array}{ll}{F}_{CQT}^{(1)}=\tfrac{3+|\,{{\rm{\Delta }}}_{1}\,|+4(|\,{z}_{1}\,|+|\,{z}_{4}\,|)}{6}, & {F}_{CQT}^{(2)}=\tfrac{3+|\,{{\rm{\Delta }}}_{1}\,|+4(|\,{z}_{2}\,|+|\,{z}_{3}\,|)}{6},\\ {F}_{CQT}^{(3)}=\tfrac{3+\sqrt{{{\rm{\Delta }}}_{2}^{2}+16{(|{z}_{1}|+|{z}_{4}|)}^{2}}}{6}, & {F}_{CQT}^{(4)}=\tfrac{3+\sqrt{{{\rm{\Delta }}}_{2}^{2}+16{(|{z}_{2}|+|{z}_{3}|)}^{2}}}{6},\end{array}$$

with Δ_2_ = (*a*_1_ − *a*_2_ + *a*_3_ − *a*_4_ − *b*_1_ + *b*_2_ − *b*_3_ + *b*_4_). Based on these results one can easily construct various mixed biseparable states useful for CQT.

***Example 1***

Let *ρ*_1_(*x*) be the one parameter *X*–matrix state described by ${a}_{1}={b}_{1}={z}_{1}=\frac{1}{2}-x$, *a*_4_ = *b*_4_ = *z*_4_ = *x* with 0 ≤ *x* ≤ 1/2 and *a*_*j*_ = *b*_*j*_ = *z*_*j*_ = 0 otherwise. In other words, the state *ρ*_1_(*x*) is just a statistical mixture of GHZ states, *ρ*_1_(*x*) = 2*x*|*GHZ*_1_〉 〈*GHZ*_1_| + (1 − 2*x*) |*GHZ*_3_〉 〈*GHZ*_3_|, where $|GH{Z}^{(1)}\rangle =\frac{1}{\sqrt{2}}(|000\rangle +|111\rangle )$ and $|GH{Z}^{(4)}\rangle =\frac{1}{\sqrt{2}}(|011\rangle +|100\rangle )$. For such state one has ${F}_{NC}({\rho }_{1}(x))=\frac{2}{3}$ and ${F}_{CQT}({\rho }_{1}(x))=\,{\rm{\max }}\{1,\frac{2}{3},\frac{5}{6},\frac{1}{2}\}=1$. This means that perfect CQT can be performed regardless of the value *x*. On the other hand, the genuine concurrence *C*_*GME*_(*ρ*_1_(*x*)) = |4*x* − 1|. For the boundary cases $x=\{0,\frac{1}{2}\}$ the genuine concurrence *C*_*GME*_(*ρ*_1_(*x*)) = 1 since the state *ρ*_1_(*x*) = |*GHZ*^(1)^〉 and *ρ*_1_(*x*) = |*GHZ*^(4)^〉, respectively. However, when $x=\frac{1}{4}$ the state *ρ*_1_(*x*) belongs to the biseparable class and it can be decomposed as ${\rho }_{1}(x)=\frac{1}{2}(|{\psi }^{+}\rangle \langle {\psi }^{+}|+|{\psi }^{-}\rangle \langle {\psi }^{-}|)$, where $|{\psi }^{\pm }\rangle =\frac{1}{2}{(\pm |0\rangle }_{C}+|1{\rangle }_{C})\otimes {(|00\rangle }_{SR}\pm |11{\rangle }_{SR})$. This outcome implies that the maximal faithfulness of CQT perform via biseparable state is equal to 1. In order to explain this fact, let us consider the above decomposition in more details. As we see, regardless of the outcome of von Neumann measurement performed in the orthogonal basis {(|0〉_*C*_ + |1〉_*C*_), (−|0〉_*C*_ + |1〉_*C*_)} the state *ρ*_1_(*x*) is reduced with equal probability to one of two maximally entangled states. However, without classical information from the controller’s side one cannot encode which Bell state it is and hence, the quantum teleportation becomes impossible. This means that the classical correlation between controller and joined “sender-receiver” subsystem is sufficient in order to either allow or forbid the controlled teleportation and no entanglement is needed. We note that the above observation can be extended to n-qubit channels if one defines $|GH{Z}_{n}^{(1)}\rangle =\frac{1}{\sqrt{2}}(|0\ldots 0\rangle +|1\ldots 1\rangle )$ and $|GH{Z}_{n}^{(4)}\rangle$ likewise. All these calculations are summarized as follows.

#### **Proposition 2**.

For n-qubit mixed states *ρ*, perfect controlled teleportation (*F*_*CQT*_(*ρ*) = 1) can be reached even if *ρ* is a statistical mixture of biseparable pure states.

***Example 2***

Let *ρ*_2_ be a mixture of W-class states $|{\psi }_{W}({\theta }_{1},{\theta }_{2})\rangle =\frac{1}{\sqrt{3}}[|GH{Z}^{(2)}\rangle -{e}^{i{\theta }_{1}}|GH{Z}^{(3)}\rangle -{e}^{i{\theta }_{2}}|GH{Z}^{(4)}\rangle ]$^42^, where all GHZ states are defined as before:

21 $$\begin{array}{rcl}{\rho }_{2}({\theta }_{1},{\theta }_{2}) & = & \frac{1}{8}[|{\psi }_{W}(\pi /3,2\pi /3)\rangle \langle {\psi }_{W}(\pi /3,2\pi /3)|\\  &  & +\,|{\psi }_{W}(5\pi /3,4\pi /3)\rangle \langle {\psi }_{W}(5\pi /3,4\pi /3)|\\  &  & +\,|{\psi }_{W}(\pi /3,5\pi /3)\rangle \langle {\psi }_{W}(\pi /3,5\pi /3)|\\  &  & +\,|{\psi }_{W}(2\pi /3,4\pi /3)\rangle \langle {\psi }_{W}(2\pi /3,4\pi /3)|\\  &  & +\,{\rm{terms}}\,{\rm{with}}\,{\rm{exchanged}}\,{\rm{arguments}}].\end{array}$$

Note that *ρ*_2_ belongs to the *X*–matrix states and hence, one can easily find ${F}_{NC}({\rho }_{2})=\frac{5}{9}$ and ${F}_{CQT}({\rho }_{2})=\frac{7}{9}$. In the same time, *C*_*GME*_(*ρ*_2_) = 0 what confirms that the biseparable states useful for CQT can be constructed starting from the W-states.

### Estimation of controlled teleportation faithfulness

Due to the variational definition of *F*_*CQT*_ in Eq. (6) it is evident that the estimation of controlled teleportation faithfulness for general mixed state remains a difficult problem which requires an optimization over all unitary transformation *U*_*C*_ and all maximally entangled states |*e*〉. It would be therefore interesting to derive a tight upper bound for the *F*_*CQT*_ solely based on the convex decomposition of the state under considerations. For this purpose, we introduce a convex-roof extension of Eq. (2) on pure states

22 $${ {\mathcal F} }_{CQT}(\rho )=\mathop{{\rm{\min }}}\limits_{\{{p}_{j},{\phi }^{(j)}\}}\,{ {\mathcal F} }_{CQT}^{\ast }(\rho )=\mathop{{\rm{\min }}}\limits_{\{{p}_{j},{\phi }^{(j)}\}}\,\sum _{j}^{m}\,{p}_{j}\frac{2+\sqrt{\tau ({\phi }^{(j)})+{{\mathscr{C}}}_{S,R}^{2}({\phi }^{(j)})}}{3},$$

where the maximum is taken over all possible pure state convex decompositions $\{{p}_{j},{\phi }^{(j)}\}$ of *ρ*, i.e. $\rho ={\sum }_{j}^{m}\,{p}_{j}|{\phi }^{(j)}\rangle \langle {\phi }^{(j)}|$, ${\sum }_{j}^{m}\,{p}_{j}=1$ and *p*_*j*_ > 0. By the very definition it is obvious that $\frac{2}{3}\le { {\mathcal F} }_{CQT}^{\ast }(\rho )\le 1$ for any decomposition of *ρ*. Based on Eq. (22) one can derive the following proposition.

#### **Proposition 3**.

For a given tripartite mixed state *ρ* the fidelity of controlled teleportation is smaller than or equal to the average conditional fidelity of any convex decomposition $\rho ={\sum }_{j}^{m}\,{p}_{j}|{\phi }^{(j)}\rangle \langle {\phi }^{(j)}|$,

23 $${F}_{CQT}(\rho )\le { {\mathcal F} }_{CQT}(\rho )\le { {\mathcal F} }_{CQT}^{\ast }(\rho ).$$

#### *Proof*.

Suppose that one has an optimal convex decomposition $\rho ={\sum }_{j}^{m}\,{p}_{j}|{\phi }^{(j)}\rangle \langle {\phi }^{(j)}|$ which minimize ${ {\mathcal F} }_{CQT}^{\ast }(\rho )$. Then, the reduced state ${\rho }_{SR}^{t}$ given by Eq. (4) can be rewritten as

24 $${\rho }_{SR}^{t}=\tfrac{{{\rm{Tr}}}_{C}[{\hat{P}}_{C}^{(t)}\,\sum _{j}^{m}\,{p}_{j}|{\phi }^{(j)}\rangle \langle {\phi }^{(j)}|{\hat{P}}_{C}^{(t)}]}{\langle t|{U}_{C}{\rho }_{C}{U}_{C}^{\dagger }|t\rangle }=\sum _{j}^{m}\,{p}_{j}\tfrac{{{\rm{Tr}}}_{C}[{\hat{P}}_{C}^{(t)}|{\phi }^{(j)}\rangle \langle {\phi }^{(j)}|{\hat{P}}_{C}^{(t)}]}{\langle t|{U}_{C}{\rho }_{C}{U}_{C}^{\dagger }|t\rangle }=\sum _{j}^{m}\,{p}_{j}|{\phi }_{SR}^{(t,j)}\rangle \langle {\phi }_{SR}^{(t,j)}|$$

where $${\hat{P}}_{C}^{(t)}={U}_{C}^{\dagger }|t\rangle \langle t|{U}_{C}\otimes {11}_{4}$$ and the last equality comes from the fact that $$|{\phi }_{SR}^{(t,j)}\rangle \langle {\phi }_{SR}^{(t,j)}|$$ is the resulting state after the orthogonal measurements performed on $$|{\phi }_{j}\rangle \langle {\phi }_{j}|$$ and hence it must be a pure state. Based on this observation and the convexity of the fully entanglement fraction (i.e. $$\langle e|{\sum }_{k}\,{p}_{k}{\sigma }_{k}|e\rangle $$$$f({\sum }_{k}\,{p}_{k}{\sigma }_{k})={{\rm{\max }}}_{e}\,$$ ≤ $${\sum }_{k}\,{p}_{k}\,{{\rm{\max }}}_{e}\,\langle e|{\sigma }_{k}|e\rangle ={\sum }_{k}\,{p}_{k}f({\sigma }_{k})$$) one achieves from Eq. (6) that

25 $$\begin{array}{rcl}{f}_{CQT}(\rho ) & = & \mathop{{\rm{\max }}}\limits_{{U}_{C}}\,[\sum _{t=0}^{1}\,\langle t|{U}_{C}{\rho }_{C}{U}_{C}^{\dagger }|t\rangle f({\rho }_{SR}^{t})]\\  & = & \mathop{{\rm{\max }}}\limits_{{U}_{C}}\,[\sum _{t=0}^{1}\,\langle t|{U}_{C}{\rho }_{C}{U}_{C}^{\dagger }|t\rangle f(\sum _{j}^{m}\,{p}_{j}|{\phi }_{SR}^{(t,j)}\rangle \langle {\phi }_{SR}^{(t,j)}|)]\\  & \le  & \mathop{{\rm{\max }}}\limits_{{U}_{C}}\,[\sum _{j}^{m}\,{p}_{j}\,\sum _{t=0}^{1}\,\langle t|{U}_{C}{\rho }_{C}{U}_{C}^{\dagger }|t\rangle f(|{\phi }_{SR}^{(t,j)}\rangle \langle {\phi }_{SR}^{(t,j)}|)]\\  & \le  & \sum _{j}^{m}\,{p}_{j}\mathop{{\rm{\max }}}\limits_{{U}_{C}}\,[\sum _{t=0}^{1}\,\langle t|{U}_{C}{\rho }_{C}{U}_{C}^{\dagger }|t\rangle f(|{\phi }_{SR}^{(t,j)}\rangle \langle {\phi }_{SR}^{(t,j)}|)]\end{array}$$

Now, following the calculations presented in ref.^20^ we have

26 $$\begin{array}{rcl}{f}_{CQT}(\rho ) & \le  & \sum _{j}^{m}\,{p}_{j}\mathop{{\rm{\max }}}\limits_{{U}_{C}}\,[\sum _{t=0}^{1}\,\langle t|{U}_{C}{\rho }_{C}{U}_{C}^{\dagger }|t\rangle f(|{\phi }_{SR}^{(t,j)}\rangle \langle {\phi }_{SR}^{(t,j)}|)]\\  & = & \sum _{j}^{m}\,{p}_{j}[\frac{1}{2}+\frac{1}{2}\sqrt{\tau ({\phi }^{(j)})+{{\mathscr{C}}}_{S,R}^{2}({\phi }^{(j)})}].\end{array}$$

Substituting this inequality into ${F}_{CQT}(\rho )=\frac{2{f}_{CQT}(\rho )+1}{3}$ one obtains ${F}_{CQT}(\rho )\le { {\mathcal F} }_{CQT}(\rho )$. Now, if an ensemble {*p*_*j*_, *φ*^(*j*)^} is chosen arbitrarily the second inequality in Eq. (26) becomes straightforward. Finally, the equality in Eq. (26) is provided (for instance) by the mixture of the GHZ states *ρ*_1_(*x*) discussed in Example 1, what ends the proof.$\square$

As an example of Proposition 3, let us consider the eigendecomposition of GHZ-symmetric states. Based on Eq. (8) one can notice that the spectral decomposition of *ρ*^*GS*^ is given by two GHZ states ${\phi }^{1,2}=|GH{Z}^{\pm }\rangle$ with the probability ${p}_{1,2}=\frac{1}{8}[1\pm 8x+4\sqrt{3}y]$ and six product states with ${p}_{3,\ldots ,8}=\frac{\sqrt{3}-4y}{8\sqrt{3}}$. Then, Eq. (10) and the right-hand side of Eq. (23) yield

27 $$\begin{array}{rcl}{F}_{CQT}({\rho }^{GS}) & = & \tfrac{1}{6}[3+4|x|+\tfrac{4\sqrt{3}}{3}y]\\  & \le  & \tfrac{1+8x+4\sqrt{3}y}{8}\times 1+\tfrac{1-8x+4\sqrt{3}y}{8}\times 1+6\tfrac{\sqrt{3}-4y}{8\sqrt{3}}\times \tfrac{2}{3}\\  & = & \tfrac{3}{4}+\tfrac{\sqrt{3}}{3}y,\end{array}$$

which is in line with Proposition 3. Naturally, for other decompositions various upper bounds of *F*_*CQT*_ are estimated. In particular, one can take an optimal decomposition $\{{p}_{j}^{opt},{\phi }_{opt}^{(j)}\}$ which minimizes the three-tangle i.e. $\tau ({\rho }^{GS})={\sum }_{j}^{m}\,{p}_{j}^{opt}\tau ({\phi }_{opt}^{(j)})$. Then, for any state beyond GHZ-class *τ*(*ρ*^*GS*^) = 0 what is equivalent to $\tau ({\phi }_{opt}^{(j)})=0$. However, such decomposition does not entails ${{\mathscr{C}}}_{S,R}^{2}({\phi }_{opt}^{(j)})=0$ what can be easily verified by applying the optimal decomposition of GHZ-symmetric states reported in ref.^36^. This observation clearly explains the failure of the *F*_*CQT*_ estimation based on “predefined” properties. From these conclusions, an important question arises whether for a tripartite mixed state there exists such an optimal ensemble {*p*_*i*_, *φ*^(*i*)^} which provides ${F}_{CQT}(\rho )={ {\mathcal F} }_{CQT}(\rho )$. Following ref.^43^, we expect that such optimal decomposition truly exists however, not for all tripartite mixed states.

#### **Proposition 4**.

Given a tripartite mixed state $\rho ={\sum }_{j}^{m}\,{p}_{j}|{\phi }^{(i)}\rangle \langle {\phi }^{(i)}|$, where $|{\phi }^{(j)}\rangle ={\sum }_{\alpha ,\beta ,\gamma =0}^{1}\,{\phi }_{\alpha \beta \gamma }^{(j)}|\alpha \beta \gamma \rangle$, then ${F}_{CQT}(\rho )={ {\mathcal F} }_{CQT}(\rho )$ if and only if (i) there exist unitary matrices ${M}_{C}^{(j)}$, ${M}_{S}^{(j)}$ and ${M}_{R}^{(j)}$ such that ${M}_{C}^{(j)}\otimes {M}_{S}^{(j)}\otimes {M}_{R}^{(j)}|{\phi }^{(j)}\rangle$ = ${\lambda }_{0}^{(j)}|000\rangle +{\lambda }_{1}^{(j)}{e}^{i{\theta }_{j}}|100\rangle$ + ${\lambda }_{2}^{(j)}|101\rangle +{\lambda }_{3}^{(j)}|110\rangle +{\lambda }_{4}^{(j)}|111\rangle$ with *λ*_*k*_ ≥ 0, 0 ≤ *θ*_*j*_ ≤ *π* and the following constraint is satisfied: ${T}_{C}^{(j)}{M}_{C}^{(j)}={T}_{C}^{(k)}{M}_{C}^{(k)}$, where $${T}_{C}^{(l)}=(\begin{array}{ll}\cos \,\tfrac{{\alpha }_{l}}{2}{e}^{i{\beta }_{l}} & -\sin \,\tfrac{{\alpha }_{l}}{2}{e}^{i{\gamma }_{l}}\\ \sin \,\tfrac{{\alpha }_{l}}{2}{e}^{-i{\gamma }_{l}} & \cos \,\tfrac{{\alpha }_{l}}{2}{e}^{-i{\beta }_{l}}\end{array})$$ and ${\alpha }_{l}=\tfrac{{\lambda }_{0}^{(l)}{\lambda }_{4}^{(l)}}{{\lambda }_{1}^{(l)}{\lambda }_{4}^{(l)}\,\cos ({\beta }_{l}+{\gamma }_{l}-{\theta }_{l})-{\lambda }_{2}^{(l)}{\lambda }_{3}^{(l)}\,\cos ({\beta }_{l}+{\gamma }_{l})}$ with 0 ≤ *β*_*l*_,*γ*_*l*_ ≤ 2*π*, (ii) for the modified state $|{\varphi }_{SR}^{(t,j)}\rangle ={\sum }_{\beta ,\gamma =0}^{1}\,\{\langle t|{U}_{C}|0\rangle {\phi }_{0\beta \gamma }^{(j)}+\langle t|{U}_{C}|1\rangle {\phi }_{1\beta \gamma }^{(j)}\}|\beta \gamma \rangle$, where ${u}_{kl}^{(j)}=\langle k|{T}_{C}^{(j)}{M}_{C}^{(j)}|l\rangle$ and *t* = {0, 1}, there exist unitary matrices ${D}_{S}^{(t,j)}$, ${D}_{R}^{(t,j)}$ which bring $({D}_{S}^{(t,j)}\otimes {D}_{R}^{(t,j)})|{\phi }_{SR}^{(t,j)}\rangle$ to the Schmidt form and ${({D}_{S}^{(t,j)})}^{\dagger }{({D}_{R}^{(t,j)})}^{\ast }={e}^{i{{\rm{\Omega }}}_{kj}^{(t)}}{({D}_{S}^{(t,k)})}^{\dagger }{({D}_{R}^{(t,k)})}^{\ast }$, where $0\le {{\rm{\Omega }}}_{kj}^{(t)}\le 2\pi$.

#### *Proof*.

In order to prove the above proposal, one has to derive sufficient conditions which provide saturation of both inequalities in Eq. (25). To do this, let us first assume that a given state *ρ* is pure, $\rho =|{\phi }^{(1)}\rangle \langle {\phi }^{(1)}|$, and $|{\phi }^{(1)}\rangle$ writes in the canonical form^28,44^ as $|{\phi }_{can}^{(1)}\rangle ={\lambda }_{0}^{(1)}|000\rangle +{\lambda }_{1}^{(1)}{e}^{i{\theta }_{1}}|100\rangle$ + ${\lambda }_{2}^{(1)}|101\rangle +{\lambda }_{3}^{(1)}|110\rangle +{\lambda }_{4}^{(1)}|111\rangle$, where *λ*_*k*_ ≥ 0, ${\sum }_{k}\,{\lambda }_{k}^{2}=1$ and 0 ≤ *θ*_1_ ≤ *π*. According to Eq. (36) the fully entanglement fraction is given by

28 $$\begin{array}{rcl}{f}_{CQT}^{(1)}(\rho ) & = & \mathop{{\rm{\max }}}\limits_{{U}_{C}}\{\mathop{{\rm{\max }}}\limits_{{V}_{0}}|\langle {\phi }_{SR}^{(0,1)}|{M}_{S}^{(1)}{V}_{0}{({M}_{R}^{(1)})}^{T}\otimes {11}_{2}|{\psi }^{+}\rangle {|}^{2}\\  &  & +\,\mathop{{\rm{\max }}}\limits_{{V}_{1}}|\langle {\phi }_{SR}^{(1,1)}|{M}_{S}^{(1)}{V}_{1}{({M}_{R}^{(1)})}^{T}\otimes {11}_{2}|{\psi }^{+}\rangle {|}^{2}\},\end{array}$$

where $|{\phi }_{SR}^{(t,1)}\rangle =({\lambda }_{0}^{(1)}{u}_{0t}+{\lambda }_{1}^{(1)}{e}^{i{\theta }_{1}}{u}_{1t})|00\rangle$ + ${\lambda }_{2}^{(1)}{u}_{1t}|01\rangle +{\lambda }_{3}^{(1)}{u}_{1t}|10\rangle +{\lambda }_{4}^{(1)}{u}_{1t}|11\rangle$, ${u}_{lt}=\langle l|{M}_{C}^{(1)}{U}_{C}^{\dagger }|t\rangle$ and the unitary matrices *M* satisfy $|{\phi }_{can}^{(1)}\rangle ={M}_{C}^{(1)}\otimes {M}_{S}^{(1)}\otimes {M}_{R}^{(1)}|{\phi }^{(1)}\rangle$. Based on the analysis presented in ref.^20^, it is known that for each term in Eq. (28) one can find such matrices *V*_*t*_ that ${{\rm{\max }}}_{{V}_{t}}\,|\langle {\phi }_{SR}^{(t\mathrm{,1)}}|{M}_{S}^{\mathrm{(1)}}{V}_{t}{({M}_{R}^{\mathrm{(1)}})}^{T}\otimes {11}_{2}|{\psi }^{+}\rangle {|}^{2}$ = $\langle t|{U}_{C}{\rho }_{C}{U}_{C}^{\dagger }|t\rangle \frac{1+{\mathscr{C}}({\rho }_{SR}^{(t\mathrm{,1)}})}{2}$, where ${\rho }_{SR}^{(t,1)}=\frac{|{\phi }_{SR}^{(t,1)}\rangle \langle {\phi }_{SR}^{(t,1)}|}{\langle t\,|{U}_{C}{\rho }_{C}{U}_{C}^{\dagger }|\,t\rangle }$. Substituting this equality into Eq. (28) and performing a straightforward maximization over *U*_*C*_ one can find that the global maximum of ${f}_{CQT}^{\mathrm{(1)}}(\rho )$ occurs iff ${M}_{C}^{(1)}{U}_{C}^{\dagger }={({T}_{C}^{(1)})}^{\dagger }$, where ${T}_{C}^{(1)}$ is given above. Fulfillment of all these conditions implies ${f}_{CQT}^{(1)}(\rho )=[1+\sqrt{\tau ({\phi }^{(1)})+{{\mathscr{C}}}_{S,R}^{2}({\phi }^{(1)})}]/2$. We note that the same result ca be found for standard form of |*φ*^(1)^〉, however in a much complicated form. In such case, the maximum of ${f}_{CQT}^{\mathrm{(1)}}(\rho )$ is reached if and only if ${U}_{C}={T}_{C}^{(1)}{M}_{C}^{(1)}$.

Now, we are in the position to prove the main result. For this purpose, let $\rho ={\sum }_{j}^{m}\,{p}_{j}|{\phi }^{(j)}\rangle \langle {\phi }^{(j)}|$, where $|{\phi }^{(j)}\rangle ={\sum }_{\alpha ,\beta ,\gamma =0}^{1}\,{\phi }_{\alpha \beta \gamma }^{(j)}|\alpha \beta \gamma \rangle$ and *m* > 1. Then, the fully entanglement fraction writes ${f}_{CQT}(\rho )={{\rm{\max }}}_{{U}_{C}}$ ${\sum }_{t=0}^{1}\,\{{{\rm{\max }}}_{{V}_{t}}\,{\sum }_{j}^{m}\,{p}_{j}|\langle {\varphi }_{SR}^{(t,j)}|{V}_{t}\otimes {11}_{2}|{\psi }^{+}\rangle {|}^{2}\}$, where the modified two-qubit state $|{\varphi }_{SR}^{(t,j)}\rangle ={\sum }_{\beta ,\gamma =0}^{1}\,\{\langle t|{U}_{C}|0\rangle {\phi }_{0\beta \gamma }^{(j)}$ $+\langle t|{U}_{C}|1\rangle {\phi }_{1\beta \gamma }^{(j)}\}|\beta \gamma \rangle$ (see Eq. (34)) and, in general, ${{\mathscr{N}}}_{SR}^{(t,j)}=\langle {\varphi }_{SR}^{(t,j)}|{\varphi }_{SR}^{(t,j)}\rangle \ne 1$. By the Schmidt decomposition, there exist unitary matrices ${D}_{S}^{(j)}$, ${D}_{R}^{(j)}$ such that $|{\tilde{\varphi }}_{SR}^{(t,j)}\rangle ={D}_{S}^{(t,j)}\otimes {D}_{R}^{(t,j)}|{\varphi }_{SR}^{(t,j)}\rangle ={{\mathscr{N}}}_{SR}^{(t,j)}\,{\sum }_{l}\,[{a}_{l}^{(t,j)}/{{\mathscr{N}}}_{SR}^{(t,j)}]|ll\rangle$ where ${a}_{l}^{(t,j)}\ge 0$ and ${\sum }_{l}\,{({a}_{l}^{(t,j)}/{{\mathscr{N}}}_{SR}^{(t,j)})}^{2}=1$. For this decomposition *f*_*CQT*_(*ρ*) can further be simplified by means of w$|\langle {\varphi }_{SR}^{(t,j)}|{V}_{t}\otimes {11}_{2}|{\psi }^{+}\rangle {|}^{2}$ = $|\langle {\tilde{\varphi }}_{SR}^{(t,j)}|{D}_{S}^{(t,j)}{V}_{t}\otimes {D}_{R}^{(t,j)}|{\psi }^{+}\rangle {|}^{2}$ = $|\langle {\tilde{\varphi }}_{SR}^{(t,j)}|{D}_{S}^{(t,j)}{V}_{t}{({D}_{R}^{(t,j)})}^{T}\otimes {11}_{2}|{\psi }^{+}\rangle {|}^{2}$, where the last equality is caused by the property $A\otimes {11}_{2}|{\psi }^{+}\rangle ={11}_{2}\otimes {A}^{T}|{\psi }^{+}\rangle$^45^. As a result one obtains

29 $${f}_{CQT}(\rho )=\mathop{{\rm{\max }}}\limits_{{U}_{C}}\,\sum _{t=0}^{1}\,\{\mathop{{\rm{\max }}}\limits_{{V}_{t}}\,\sum _{j}^{m}\,{p}_{j}|\langle {\tilde{\varphi }}_{SR}^{(t,j)}|{D}_{S}^{(t,j)}{V}_{t}{({D}_{R}^{(t,j)})}^{T}\otimes {11}_{2}|{\psi }^{+}\rangle {|}^{2}\}.$$

Now, the first inequalities in Eq. (25) is saturated if and only if ${f}_{CQT}(\rho )={f}_{CQT}^{\ast }(\rho )$, where

30 $${f}_{CQT}^{\ast }(\rho )=\mathop{{\rm{\max }}}\limits_{{U}_{C}}\,\sum _{j}^{m}\,{p}_{j}\{\sum _{t=0}^{1}\,\mathop{{\rm{\max }}}\limits_{{V}_{(t,j)}}|\langle {\tilde{\varphi }}_{SR}^{(t,j)}|{D}_{S}^{(t,j)}{V}_{(t,j)}{({D}_{R}^{(t,j)})}^{T}\otimes {11}_{2}|{\psi }^{+}\rangle {|}^{2}\}.$$

Following ref.^46^ it is known that ${f}_{CQT}^{\ast }(\rho )$ is maximized with respect to *V*_(*t*,*j*)_ when ${D}_{S}^{(t,j)}{V}_{(t,j)}{({D}_{R}^{(t,j)})}^{T}={e}^{i{{\rm{\Omega }}}^{(t,j)}}{11}_{2}$ i.e. ${V}_{(t,j)}={e}^{i{{\rm{\Omega }}}^{(t,j)}}{({D}_{S}^{(t,j)})}^{\dagger }{({D}_{R}^{(t,j)})}^{\ast }$. Base on the result, ${f}_{CQT}(\rho )={f}_{CQT}^{\ast }(\rho )$ iff for any 1 ≤ *j*, *k* ≤ *m* one has *V*_*t*_ = *V*_(*t*,*j*)_ = *V*_(*t*,*k*)_ what implies ${({D}_{S}^{(t,j)})}^{\dagger }{({D}_{R}^{(t,j)})}^{\ast }={e}^{i({{\rm{\Omega }}}^{(t,k)}-{{\rm{\Omega }}}^{(t,j)})}{({D}_{S}^{(t,k)})}^{\dagger }{({D}_{R}^{(t,k)})}^{\ast }$. Similarly, the second inequalities in Eq. (25) is saturated iff ${f}_{CQT}^{\ast }(\rho )={f}_{CQT}^{\ast \ast }(\rho )$, where

31 $${f}_{CQT}^{\ast \ast }(\rho )=\sum _{j}^{m}\,{p}_{j}\{\mathop{{\rm{\max }}}\limits_{{U}_{C}^{j}}\,\sum _{t=0}^{1}\,\mathop{{\rm{\max }}}\limits_{{V}_{t}}|\langle {\tilde{\varphi }}_{SR}^{(t,j)}|{D}_{S}^{(t,j)}{V}_{t}{({D}_{R}^{(t,j)})}^{T}\otimes {11}_{2}|{\psi }^{+}\rangle {|}^{2}\},$$

As it is described above, the maximum of ${f}_{CQT}^{\ast \ast }(\rho )$ occurs when ${U}_{C}^{j}={T}_{C}^{(j)}{M}_{C}^{(j)}$. Consequently, ${f}_{CQT}^{\ast }(\rho )={f}_{CQT}^{\ast \ast }(\rho )$ iff for any 1 ≤ *j*, *k* ≤ *m* one has ${U}_{C}={U}_{C}^{j}={U}_{C}^{j}$ i.e. when ${T}_{C}^{(j)}{M}_{C}^{(j)}={T}_{C}^{(k)}{M}_{C}^{(k)}$. Finally, for ${U}_{C}={T}_{C}^{(j)}{M}_{C}^{(j)}$ the fully entanglement fraction ${f}_{CQT}^{\ast \ast }(\rho )={\sum }_{j}^{m}\,{p}_{j}\tfrac{1+\sqrt{\tau ({\phi }^{(1)})+{{\mathscr{C}}}_{S,R}^{2}({\phi }^{(1)})}}{2}$ and hence, from Eq. (6) one achieves ${F}_{CQT}(\rho )={ {\mathcal F} }_{CQT}(\rho )$, what ends the proof.$\square$

It is worth mentioning that Proposition 4 gives the condition when *f*_*CQT*_(*ρ*) and hence *F*_*CQT*_(*ρ*) fulfills the convex-roof measure. Furthermore, *f*_*CQT*_(*ρ*) in Eq. (31) is a special extension of the bipartite counterpart^43^. Following the interpretation of fully entanglement fraction as a distance between analyzed state and maximally entangled states, it is clear from Eq. (31) that the larger *f*_*CQT*_(*ρ*), the closer $|{\tilde{\varphi }}_{SR}^{(t,j)}\rangle$ and maximally entangled states are.

## Conclusions

We have investigated the performance of the controlled quantum teleportation protocol via three-qubit mixed state channels. In particular, we have analyzed the nontrivial family of high-rank mixed states called the GHZ-symmetric states. For such states the detection of various entanglement classes can be carried out analytically. Therefore, the GHZ-symmetric states represent good candidates for the discussion on the usefulness of tripartite entanglement states as a necessary resource for quantum CQT protocol. For this purpose we have analyzed the fidelity of the CQT and shown that this protocol can be performed not only through mixed states that belong to the GHZ-class and W-class but also via mixed biseparable states. This suggests a counterintuitive fact since for pure-state channels there is no biseparable state suitable for CQT. As a consequence, none of the tripartite entanglement (neither GHZ-class nor W-class) can be considered as a necessary resource for controlled teleportation protocol. The results given here also implies a conclusion that the faithfulness of controlled teleportation is more robustness against noise than the tripartite entanglement. This observation is illustrated by the analysis of three-qubit Werner states. Finally, we have proven that the necessary (but not sufficient) condition for CQT is the localizable entanglement. In particular, the localizable concurrence plays the same role in CQT as bipartite concurrence in the standard teleportation protocol. Further studies of the three-qubit X-states have revealed that our observations are non-negligible and also crucial for proper explanation of CQT protocol. Specifically, we have shown that a statistical mixture of biseparable states can be suitable for the perfect faithfulness of CQT. In this particular example no entanglement between controller’s qubit and the rest of the system exists. Despite of that the controller’s permission initiates the protocol what entails that the classical correlation are sufficient to authorized the controller’s power. Importantly, above results open new possible ways of implementation of CQT lowering requirements for a state preparation and preservation. A particular example of the CQT implementation based on the quantum dots system has been recently published in^47^. Finally, we have investigated the upper limitation of *F*_*CQT*_ with respect to an arbitrary convex decomposition of the analyzed state. We have shown that for a given mixed state the teleportation fidelity is always smaller than or equal to the mean fidelity of its convex decomposition. Furthermore, we have establish sufficient conditions when the equality in this proposition takes place.

Even though we have analyzed the three-qubit system, it is of great importance to mention that the main results of the paper are extended to general N-qubit systems and hence, motivate further research directions. Specifically, it is known, that the localizable entanglement restricted to projective von Neuman measurements (PM), positive operator-valued measures (POVM) and general LOCC measures satisfies the relation ${{\mathscr{C}}}_{L}^{PM}(\rho )\le {{\mathscr{C}}}_{L}^{POVM}(\rho )\le {{\mathscr{C}}}_{L}^{LOCC}(\rho )$^40^. For that reason, one may attempt to determine the amount the faithfulness of controlled teleportation through N-qubit channels when the controller is allowed to perform various kinds of measurements. For what types of N-qubit states are local measurements sufficient for CQT, and when more general measurements can enhance the fidelity of teleportation.

## Method

### Fully entanglement fraction for CQT

By the definition of the reduced state ${\rho }_{SR}^{t}=$ $\tfrac{{\sum }_{q=0}^{1}\,[\langle q\,|{U}_{C}^{\dagger }|\,t\rangle \langle t\,|{U}_{C}\otimes {11}_{4}\rho {U}_{C}^{\dagger }|\,t\rangle \langle t\,|{U}_{C}|\,q\rangle \otimes {11}_{4}]}{\langle t\,|{U}_{C}{\rho }_{C}{U}_{C}^{\dagger }|\,t\rangle }$ and hence,

32 $$\begin{array}{rcl}f({\rho }_{SR}^{t}) & = & \mathop{{\rm{\max }}}\limits_{{e}^{t}}\langle {e}^{t}|{\rho }_{SR}^{t}|{e}^{t}\rangle \\  & = & \mathop{{\rm{\max }}}\limits_{{e}^{t}}\,\tfrac{{\sum }_{q=0}^{1}\,[\langle q\,|{U}_{C}^{\dagger }|\,t\rangle \langle t\,|{U}_{C}\otimes \langle {e}^{t}\,|\rho {U}_{C}^{\dagger }|\,t\rangle \langle t\,|{U}_{C}|\,q\rangle \otimes |\,{e}^{t}\rangle ]}{\langle t\,|{U}_{C}{\rho }_{C}{U}_{C}^{\dagger }|\,t\rangle }\\  & = & \mathop{{\rm{\max }}}\limits_{{e}^{t}}\,\sum _{j}^{m}\,{p}_{j}\tfrac{\langle {\phi }^{(j)}\,|[{U}_{C}^{\dagger }|\,t\rangle \otimes |\,{e}^{t}\rangle ]\,[\langle t\,|{U}_{C}\otimes \langle {e}^{t}\,|]|{\phi }^{(j)}\rangle }{\langle t\,|{U}_{C}{\rho }_{C}{U}_{C}^{\dagger }|\,t\rangle }\\  & = & \mathop{{\rm{\max }}}\limits_{{e}^{t}}\,\sum _{j}^{m}\,{p}_{j}\tfrac{|\langle {\phi }^{(j)}\,|[{U}_{C}^{\dagger }\otimes {11}_{4}][|\,t\rangle \otimes |\,{e}^{t}\rangle ]{|}^{2}}{\langle t\,|{U}_{C}{\rho }_{C}{U}_{C}^{\dagger }|\,t\rangle }\end{array}$$

where we have used $\rho ={\sum }_{j}^{m}\,{p}_{j}|{\phi }^{(j)}\rangle \langle {\phi }^{(j)}|$ and the fact that ${\sum }_{q=0}^{1}\,\langle q|{U}_{C}^{\dagger }|t\rangle \langle t|{U}_{C}|q\rangle =1$ for *t* = 0, 1. Let us now derive the fully entanglement fraction for CQT, *f*_*CQT*_(*ρ*), in two cases: standard and canonical parametrization of |*φ*^(*j*)^〉.

(i) Any pure state $|{\phi }^{(j)}\rangle ={\sum }_{\alpha ,\beta ,\gamma =0}^{1}\,{\phi }_{\alpha \beta \gamma }^{(j)}|\alpha \beta \gamma \rangle ={\sum }_{\alpha =0}^{1}\,|\alpha \rangle |{\phi }_{\alpha }^{(j)}\rangle$, where $|{\phi }_{\alpha }^{(j)}\rangle ={\sum }_{\beta ,\gamma =0}^{1}\,{\phi }_{\alpha \beta \gamma }^{(j)}|\alpha \beta \gamma \rangle$. Based on this parametrization one gets $|\langle {\phi }^{(j)}|[{U}_{C}^{\dagger }\otimes {11}_{4}][|t\rangle \otimes |{e}^{t}\rangle ]{|}^{2}$ = ${\sum }_{\alpha =0}^{1}\,\langle \alpha |{U}_{C}^{\dagger }|t\rangle \langle {\phi }_{\alpha }^{(j)}|{e}^{t}\rangle =\langle {\varphi }_{SR}^{(t,j)}|{e}^{t}\rangle$, where we have introduced $|{\varphi }_{SR}^{(t,j)}\rangle ={\sum }_{\alpha =0}^{1}\,\langle t|{U}_{C}|\alpha \rangle |{\phi }_{\alpha }^{(j)}\rangle$ = ${\sum }_{\beta ,\gamma =0}^{1}\,\{\langle t|{U}_{C}|0\rangle {\phi }_{0\beta \gamma }^{(j)}+\langle t|{U}_{C}|1\rangle {\phi }_{1\beta \gamma }^{(j)}\}|\beta \gamma \rangle$. Now, the entanglement fraction in Eq. (32) is given by
  33 $$f({\rho }_{SR}^{t})=\mathop{{\rm{\max }}}\limits_{{e}^{t}}\,\sum _{j}^{m}\,{p}_{j}\frac{|\langle {\varphi }_{SR}^{(t,j)}|[{V}_{t}\otimes {11}_{2}|{\psi }^{+}\rangle {|}^{2}}{\langle t|{U}_{C}{\rho }_{C}{U}_{C}^{\dagger }|t\rangle },$$
  where $|{e}^{t}\rangle ={V}_{t}\otimes {11}_{2}|{\psi }^{+}\rangle$ with *V*_*t*_ being a unitary matrix and |*ψ*^+^〉 the Bell state^33^. Substituting Eqs (33) to (6) one obtains
  34 $$\begin{array}{rcl}{f}_{CQT}(\rho ) & = & \mathop{{\rm{\max }}}\limits_{{U}_{C}}\,\{\mathop{{\rm{\max }}}\limits_{{V}_{0}}\,\sum _{j}^{m}\,{p}_{j}|\langle {\varphi }_{SR}^{(0,j)}|{V}_{0}\otimes {11}_{2}|{\psi }^{+}\rangle {|}^{2}\\  &  & +\,\mathop{{\rm{\max }}}\limits_{{V}_{1}}\,\sum _{j}^{m}\,{p}_{j}|\langle {\varphi }_{SR}^{(t,j)}|{V}_{1}\otimes {11}_{2}|{\psi }^{+}\rangle {|}^{2}\}.\end{array}$$
(ii) On the other hand, any three-qubit pure state can also be written in a canonical form as $|{\phi }_{can}^{(j)}\rangle ={M}_{C}^{(j)}\otimes {M}_{S}^{(j)}\otimes {M}_{R}^{(j)}|{\phi }^{(j)}\rangle$ where ${M}_{C}^{(j)}$ denotes 2 × 2 unitary matrix and $|{\phi }_{can}^{(j)}\rangle ={\lambda }_{0}^{(j)}|000\rangle +{\lambda }_{1}^{(j)}{e}^{i{\theta }_{j}}|100\rangle$ + ${\lambda }_{2}^{(j)}|101\rangle +{\lambda }_{3}^{(j)}|110\rangle +{\lambda }_{4}^{(j)}|111\rangle$^28,44^. Then, the expression $|\langle {\phi }^{(j)}|[{U}_{C}^{\dagger }\otimes {11}_{4}][|t\rangle \otimes |{e}^{t}\rangle ]{|}^{2}$ = $|\langle {\phi }_{can}^{(j)}|[{M}_{C}^{(j)}{U}_{C}^{\dagger }\otimes {M}_{S}^{(j)}{V}_{t}\otimes {M}_{R}^{(j)}][|t\rangle \otimes |{\psi }^{+}\rangle ]{|}^{2}$ = $|\langle {\phi }_{can}^{(j)}|[{M}_{C}^{(j)}{U}_{C}^{\dagger }\otimes {M}_{S}^{(j)}{V}_{t}{({M}_{R}^{(j)})}^{T}\otimes {11}_{2}][|t\rangle \otimes |{\psi }^{+}\rangle ]{|}^{2}$, where $|{e}^{t}\rangle ={V}_{t}\otimes {11}_{2}|{\psi }^{+}\rangle$ as before and the last equality is caused by the property $A\otimes {11}_{2}|{\psi }^{+}\rangle ={11}_{2}\otimes {A}^{T}|{\psi }^{+}\rangle$^45^. Further simplifications of the last expression yield

35 $$f({\rho }_{SR}^{t})=\mathop{{\rm{\max }}}\limits_{{V}_{t}}\,\sum _{j}^{m}\,{p}_{j}\frac{|\langle {\phi }_{SR}^{(t,j)}|{M}_{S}^{(j)}{V}_{t}{({M}_{R}^{(j)})}^{T}\otimes {11}_{2}|{\psi }^{+}\rangle {|}^{2}}{\langle t|{U}_{C}{\rho }_{C}{U}_{C}^{\dagger }|t\rangle },$$

where $|{\phi }_{SR}^{(t,j)}\rangle =({\lambda }_{0}^{(j)}{u}_{0t}+{\lambda }_{1}^{(j)}{e}^{i{\theta }_{j}}{u}_{1t})|00\rangle$ + ${\lambda }_{2}^{(j)}{u}_{1t}|01\rangle +{\lambda }_{3}^{(j)}{u}_{1t}|10\rangle +{\lambda }_{4}^{(j)}{u}_{1t}|11\rangle$ and ${u}_{kl}=\langle k|{M}_{C}^{(j)}{U}_{C}^{\dagger }|l\rangle$. Substituting Eqs (35) to (6) one gets

36 $$\begin{array}{rcl}{f}_{CQT}(\rho ) & = & \mathop{{\rm{\max }}}\limits_{{U}_{C}}\,\{\mathop{{\rm{\max }}}\limits_{{V}_{0}}\,\sum _{j}^{m}\,{p}_{j}|\langle {\phi }_{SR}^{(0,j)}|{M}_{S}^{(j)}{V}_{0}{({M}_{R}^{(j)})}^{T}\otimes {11}_{2}|{\psi }^{+}\rangle {|}^{2}\\  &  & +\,\mathop{{\rm{\max }}}\limits_{{V}_{1}}\,\sum _{j}^{m}\,{p}_{j}|\langle {\phi }_{SR}^{(1,j)}|{M}_{S}^{(j)}{V}_{1}{({M}_{R}^{(j)})}^{T}\otimes {11}_{2}|{\psi }^{+}\rangle {|}^{2}\}.\end{array}$$
